# The Role of Long-Chain Fatty Acids in Inflammatory Bowel Disease

**DOI:** 10.1155/2019/8495913

**Published:** 2019-11-03

**Authors:** Chunxiang Ma, Reshma Vasu, Hu Zhang

**Affiliations:** ^1^Department of Gastroenterology, West China Hospital, Sichuan University, Chengdu, China; ^2^Centre for Inflammatory Bowel Disease, West China Hospital, Sichuan University, Chengdu, China; ^3^West China School of Medicine, Sichuan University, Chengdu, China

## Abstract

Inflammatory bowel disease (IBD) is a complicated disease involving multiple pathogenic factors. The complex relationships between long-chain fatty acids (LCFAs) and the morbidity of IBD drive numerous studies to unravel the underlying mechanisms. A better understanding of the role of LCFAs in IBD will substitute or boost the current IBD therapies, thereby obtaining mucosal healing. In this review, we focused on the roles of LCFAs on the important links of inflammatory regulation in IBD, including in the pathogen recognition phase and in the inflammatory resolving phase, and the effects of LCFAs on immune cells in IBD.

## 1. Introduction

Inflammatory bowel disease (IBD), comprising Crohn's disease (CD) and ulcerative colitis (UC), is a chronic, remitted, and disabled inflammatory condition. Although several lines of evidence decipher the associated risk factors for IBD, it is still difficult to interpret the exact pathogenesis. However, regardless of the underlying etiopathogenesis, the sustained inflammation in the intestine does represent an important pathological feature for IBD [1]. Healthy intestinal mucosa depends on a complex inflammation-related equilibrium. Once the counterbalance is discomposed, the excessive proinflammatory cytokines will accelerate IBD progression. Therefore, using anti-inflammatory treatments to counteract the overproduction of intestinal proinflammatory cytokines represents the primary therapeutic approaches to control IBD aggravation. Currently, the ambition of the treatment scheme in IBD is to obtain mucosal healing [2]. The existing drug regimens to achieve this aim in IBD mainly include steroids, immunosuppressants, and biologics. Nonetheless, aside from the expensive medical expenditure, the side effects carried by these drugs also motivate doctors to develop other cheaper and more available therapeutic approaches. These emerging treatments are expected to substitute or boost the current IBD therapies.

Long-chain fatty acids (LCFAs) have a reciprocal relationship with IBD. With industrialization development, the morbidity of IBD raises dramatically in developing countries, which could be ascribed to a marked shift in the dietary mode to a certain extent [3]. It is found that the increased incidence of IBD synchronizes with a western-oriented alimentary habit (a higher ratio of n-6/n-3 long-chain polyunsaturated fatty acids (PUFAs) and an abundance of saturated long fatty acids) [4]. To date, mounting epidemiology evidence indicates that LCFAs are crucial for the etiology of IBD. For instance, a prospective study analysis reveals that a higher ratio of n-3/n-6 long-chain PUFA intake keeps an inverse association with the IBD onset [5]. The beneficial role to maintain IBD remission with such dietary intervention is equal to the role observed in another prospective analysis [6]. Beyond the epidemiological relation, LCFAs are implicated in modulating intestinal damage on both the gross lesion and histopathological change. Hassan et al. [7] reported that the supplement of n-3 PUFAs could mitigate intestinal hyperemia, ulcerations, and necrosis in a relapsed colitis model, 2,4,6-trinitrobenzene sulfonic acid- (TNBS-) induced rat colitis. Moreover, the fact that n-3 PUFAs restore mucosal architecture and ulceration was also manifested in an acute enteritis model, dextran sulfate sodium- (DSS-) induced mouse colitis [8]. In addition, gross intestinal health often is reflected by villus and crypt construction, which are essential for the protection of the intestinal epithelial barrier. Consistent with the mentioned findings, the supplementation of n-3 PUFAs was shown to ameliorate intestinal morphologic damage and increase villus height on a piglet model challenged by lipopolysaccharides (LPS) [9]. In contrast to n-3 PUFAs, n-6 PUFAs produce a significantly lower villus height than the control group, rather than improve mucosal morphology in total parenteral nutrition- (TPN-) related gut barrier impairment, indicating that n-6 PUFAs have a deteriorated effect on intestinal defense [10]. The detrimental movement of n-6 PUFAs on the gut is further replicated in the IBD animal model, in which n-6 PUFA application primes extensive depletion of goblet cells and overwhelming infiltration of leukocytes in the intestinal mucosa [11]. In conclusion, the wealth of evidence supports that various LCFAs have complex effects on intestinal inflammation and IBD.

Although a number of previous researches regarding LCFAs have been published, most investigations just deemed LCFAs as an essential nutrient in the regulation of energy metabolism. Nowadays, growing appreciations are starting to focus on their roles on inflammatory regulation in many diseases and are making endeavors to unravel the underlying mechanisms. In this review, we will provide a comprehensive insight into the mechanisms, by which LCFAs have an important role in regulating intestinal inflammation, especially in IBD, aimed at holding potential as targets for novel curative treatment.

## 2. LCFA Derivatives

LCFAs are defined as a sort of fatty acids with a carbon chain length of 13 to 21 carbons [12]. According to the number of double bonds, LCFAs could be again subdivided into saturated (no double bond), monounsaturated (one double bond), or polyunsaturated fatty acids (more than two double bonds). Accumulating studies demonstrate that oleic acid of monounsaturated fatty acids (MUFAs) imbues a beneficial effect on intestinal inflammation in IBD [13, 14]. However, the roles of PUFAs on intestinal inflammation tend to be perplexing. Eicosapentaenoic acid (EPA) and docosahexaenoic acid (DHA), rich in fish oil, are precursors for n-3 PUFAs and are categorized with n-6 PUFA-derived arachidonic acid (AA) as important lipid mediators in immune regulation and inflammation. Conventionally, n-6 PUFAs are involved in the proinflammatory effect because linoleic acid (LA, n-6 PUFAs) can be metabolized into AA, which by the cyclooxygenase (COX) pathway form prostaglandins (PGs), thromboxanes (TXs), and leukotrienes (LTs) (a series of inflammatory mediators with pleiotropic functions). In contrast, n-3 PUFAs seem to facilitate inflammatory regulation, for which *α*-linolenic acid (ALA, n-3 PUFAs) can be converted into EPA and DHA, in which the two sorts of PUFAs compete with the synthesis of each one [15]. Therefore, the disruption of a suitable n-6/n-3 PUFA rate will favor ongoing inflammation. It is not probable to only consider the role of one fatty acid within the inflammatory process without considering the functions of another fatty acid.

Inflamed colonic mucosa in UC patients is characterized by a higher concentration of AA and a lower concentration of the EPA [15]. The higher content of AA competes with the same mechanism shared by n-3 PUFAs and then enhances LA-associated proinflammatory components, which is consistent with the inflammatory severity of intestinal mucosa. Additionally, studies from TNBS-induced mouse colitis have also shown that an n-3 PUFA diet decreases COX-2 expression and LTB4 production in the colon [16]. It is not hard to understand that any exacerbated gut inflammatory in IBD tips the inflammatory homeostasis shifting to the proinflammatory side. Moreover, plant-derived oils rich in ALA, rather than fish-derived ALA, were supported on the TNBS rat model with a prominent effect of downregulating COX2 mRNA levels rather than fish-derived ALA [17]. Since plant-derived n-3 PUFAs are a cheaper and more accessible source and are superior to reduce intestinal inflammation, this type of n-3 PUFAs should be prescribed widely in IBD patients.

## 3. Regulation of LCFAs in the Inflammatory Process

The key steps of the inflammatory process can be generally classified as three interconnected and sequential phases: (i) the pathogen recognition phase, in which pathogens penetrate the epithelial barrier or bond to receptors; (ii) the mobilization phase, in which immune cells immigrate from blood to the tissue, a process promoted by adhesion molecules and chemoattractants; and (iii) the resolution phase, in which harmful agents are eliminated by anti-inflammation mediators. The successful inflammatory response is crucial to control, or at least limit aggression, and aid to the repair of intestinal injury. As the discussion below, LFCAs are responsible for each of these phases to participate in the inflammatory process of IBD.

### 3.1. Effects of LCFAs in the Pathogen Recognition Phase

The pathogen recognition phase is part of intestinal immune response, which depends on the innate sensors on the intestinal epithelial cell, such as pattern recognition receptors (PRRs). They quickly recognize pathogen components and systemically and/or locally influence inflammatory transcription factors. A tailored activation of transcription factors is vital to intestinal barrier function. LCFAs involve in this phage to regulate intestinal inflammation in IBD (Table 1).

The intestinal barrier is the first line of gut defense against bacteria and other microorganisms. Intestinal epithelial cells and tight junctions in between them shape a physical barrier to contact with extrinsic factors as well as to maintain tissue homeostasis. Tight junction proteins, such as occludin, claudins, zona occludens- (ZO-) 1, and junctional adhesion molecules, are proven to be the main component of tight junctions (TJs). Aside from the physical barrier, the chemical barrier, constituted by intestinal mucins (MUCs), containing antimicrobial peptides and secretory immunoglobulins (sIg), is also important in the prevention of intestinal pathogen invasion [18]. In IBD pathogenesis, the susceptibility to intestinal inflammation and the severity of gut lesions will be sharpened due to the dysfunction of TJ molecules or mucus layer. Recently, dietary DHA and EPA have been demonstrated to maintain intestinal barrier function in IL-10-deficient mice by rescuing the expression of occludin and ZO-1 [19]. The protective role could attain optimization by application with phospholipid DHA to restore intestinal barrier [20]. Additionally, this favorable effect of n-3 PUFA acts in a concentration-dependent manner. Various concentrations of n-3 PUFAs were used by Beguin et al. [21] to incubate Caco-2 cells, a model of human intestinal barrier. The 30 mM DHA did not affect any component of intestinal barriers, while when the concentration reached 150 mM, ZO-1 intensity was increased. Incubation with n-6 PUFAs lowering the intensity of occludin also was found in this research. To determine the effect of LCFAs on MUC2 production, a subsequent team [22] applied a large scale of fatty acids, including saturated LCFAs, MUFAs, and PUFAs, to stimulate human colonic mucus-secreting HT29-MTX cells in vitro and rats in vivo. Only saturated LCFAs (palmitic acid and palm oil) were involved in the upregulation of MUC2 production in two kinds of experiments; the other types of fatty acids led to MUC2 reduction. Collectively, DHA and EPA serve as protectors for the gut barrier in IBD due to their ability to recover the TJ-related elements, together with certain saturated LCFAs strengthening MUC2 secretion. In contrast, n-6 PUFAs impair the structure to facilitate intestinal inflammation. Considering the difference between the animal model and human body, further researches are required to explore these mechanisms in IBD patients and identify the optimum concentration.

PRRs receive the information from various pathogens and danger sensors and initiate intestinal inflammation. As members of PRRs, toll-like receptors (TLRs) play crucial roles in immune response by recognizing the accessory structures of pathogen molecules. The associations between TLRs and LCFAs in intestinal inflammation have been confirmed in experimental colitis. Evidence indicates that n-3 PUFAs and n-9 PUFAs, respectively, upregulate the TLR-2 and TLR-4 genes of TNBS-induced colitis, while n-6 PUFAs influence high-mobility group box 1 (HMGB1), a reactivator of TLR gene [16]. An ALA-rich diet also activates genes that encode inhibitors of TLR signaling, IL-1 receptor-associated kinase 1 (IRAK1), which is a negative regulator of TLR and IL-1 receptor signaling [23]. On the transcriptional level, this supplement could not only inhibit the mRNA expression of TLR4 but also regulate the downstream inflammatory cytokines in the colitis model, containing the downregulation of proinflammatory cytokines IL-1*β*, IL-6, and TNF-*α* and the upregulation of anti-inflammatory cytokine IL-10. It is well accepted that a superiority level of IL-1*β*, IL-6, TNF-*α*, and IL-8 means a common feature of many inflammatory conditions, including IBD [24]. What is more, this research indicates that n-3 PUFAs inhibit transcription factors downstream of the TLR-associated factor 6 pathway. Similar to TLR4, the nucleotide-binding oligomerisation domain (NOD) family, another PRR, participates in the modulation of inflammatory response through nuclear factor kappa B (NF-*κ*B) activation and inflammasome formation, in which the later component leads to the maturation of proinflammatory cytokines, such as IL-1*β* and IL-18 [25, 26]. Oil-rich ALA is indicated to decrease the abundance of mRNA of NOD on a model of intestinal injury, subsequent to exerting protection for intestinal integrity and barrier function [9]. These researches indicate that n-3 PUFAs confer intestinal inflammation with a protective effect for downregulating TLR/NOD pathways. In contrast, saturated fatty acid and n-6 PUFAs fail to improve intestinal inflammation, even disrupt the intestinal barrier by upregulating TLR/NOD pathways [27]. In addition, TLR4 has been reported to have a higher expression on intestinal epithelial cells (IECs) in IBD patients than control individuals [28], along with the fact that NOD has been the first identified susceptibility gene for IBD. It suggests that a high content of n-3 PUFAs virtually exerts beneficial effects on IBD-related inflammation via TLR4/NOD signaling pathways. Some noteworthiness should be paid on the conflicting effect of TLR blocker on intestinal inflammation. TLR4 blockers are declared to have beneficial effects on acute gut inflammation, while it is known as an impeder for intestinal mucosal healing in DSS-induced colitis [29]. Acting as the agonist or antagonist for TLR/NOD, LCFAs are needed to be further studied in IBD regarding long-term prognosis.

NF-*κ*B is an important component of TLR/NOD signaling pathways. Typically, the inactive NF-*κ*B is anchored in the cytoplasm with I*κ*B (inhibitor of NF-*κ*B), which impedes NF-*κ*B bonding to its nuclear localization sequence (NLS). Once stimulated, I*κ*B will be phosphorylated, and the transcription of the targeting gene will be initiated following NF-*κ*B entering the nucleus [30]. Furthermore, this paradigm will augment the levels of proinflammatory cytokines to cause severe intestinal inflammation in IBD, including COX-2, IL-1b, and IL-6 in acute inflammatory status, as well as CXCL12 and CXCL13 in the chronic inflammatory state [31, 32]. In fact, LFCAs can act on the expression of NF-*κ*B on immunity cells to affect the inflammatory launching. Fish oil-fed mice are displayed with a decreased production of TNF, IL-1*β*, and IL-6 on endotoxin-stimulated macrophages [33, 34], which is beneficial to IBD intestinal inflammation. Besides, ALA, EPA, and DHA were demonstrated to reduce the expression of TNF-*α*, LTB4, and COX-2 by inhibiting NF-*κ*B activity in rats with TNBS-induced colitis [7]. In contrast, oleic acid (n-9 PUFAs) is not documented to exert a suppressive effect on colitis activity through this pathway [35]. Furthermore, after adding n-3 PUFA to conventional treatment (5-ASA), a lower NF-*κ*B activation can be observed in TNBS-induced colitis, which provides a cogent explanation for the favorable effects of n-3 PUFAs on intestinal inflammation. However, whether the addition of n-3 PUFA can assist the curative effects of other IBD standard treatments is not yet revealed. So, numerous potential investigations and studies are warranted to be performed in the future.

Peroxisome proliferator-activated receptor- (PPAR-) *γ* is another component of the TLR/NOD signaling pathway. As a transcription factor, PPAR-*γ* interferes with the translocation of NF-*κ*B to the nucleus and then executes an indispensable anti-inflammatory role. The impaired PPAR-*γ* level was confirmed on intestinal mucosa both in IBD patients and animal models. At present, numerous PPAR-*γ* agonists have been applied in the clinical practice to treat IBD patients, for example, the commonly used 5-aminosalicylic acid (a known PPAR-*γ* agonist) [36]. The LCFAs have also emerged as important regulators of PPAR-*γ* expression, providing another important treatment option for IBD patients. Conjugated linoleic acid (CLA) can ameliorate DSS colitis through the repression of TNF-*α* expression and NF-*κ*B activation and the induction of PPAR-*γ* [37]. Another study presumed that CLAs, as a supplement with probiotics (VSL#3), could be more effective to control intestinal inflammation through the activation of PPAR-*γ* [38]. To further verify the regulation mechanism of PPAR-*γ* on colitis, Bassaganya-Riera et al. [39] found that the beneficial effect of CLA and VSL#3 in mice with DSS colitis depended on PPAR-*γ* in myeloid cells. The loss of PPAR-*γ* in myeloid cells would abrogate such protective effect. CLA was also shown in the clinical trial to ameliorate intestinal inflammation. In an open-label study, after a period of 12 weeks of administration with CLA, PPAR-*γ* on peripheral blood CD4+ and CD8+ T cell in mild to moderately active CD patients were conspicuously repressed, along with a prominent descent of the CD activity index from 245 to 187 [40]. Regarding the effectiveness of LCFAs in regulating PPAR-*γ* expression, Marion-Letellier et al. [41] investigated that DHA and EPA could even attain the similar role of troglitazone on PPAR-*γ* in Caco-2 cells. These findings show that the induction and activation of CLAs, DHA and EPA, which act as PPAR-*γ* agonists, indeed contribute to the abrogation of intestinal inflammation in IBD.

### 3.2. Effects of LCFAs on Immunity Cells

Inflammatory mediators produced in the acute phase, such as TNF-*α* and IL-1*β*, upregulate the transcription of chemokine genes, which subsequently recruit immune cells from intravascular blood into inflamed areas. IBD is a complex disease accompanied by prominent infiltration of inflammatory cells, including T lymphocytes, macrophages, neutrophils, mast cells, and plasma cells. LCFAs have been implicated in the regulation of immune cells in IBD (Table 2).

Neutrophils are the first type of inflammatory cells to transmigrate endothelial cells and infiltrate to inflammatory foci, where neutrophils differentiate into polymorphonuclear (PMN) and macrophages. The transmigration process is promoted by the formation of chemokine gradients and the upregulation of adhesion molecules, in which intercellular adhesion molecule-1 (VCAM-1) and vascular cell adhesion molecule-1 (ICAM-1) are key molecules. As the extent of PMN infiltration in intestinal mucosa exhibits a correlation with the severity of IBD, weakening the production of chemoattractants and adhesion molecules is an ideal approach to control IBD intestinal lesions [42]. Cumulative studies demonstrate the anti-inflammatory properties of LCFAs acting in this manner on experimental colitis models. The n-6 PUFAs downregulate the expression of chemoattractant production C-X-C motif ligand-1 (CXCL1) and C-C motif ligand-2 (CCL2) on intestinal ischemia/reperfusion injury [43], as well as n-3 PUFAs inhibit chemokine production such as interleukin-8 (also known as CXCL8) [41]. Meanwhile, the beneficial effects of n-3 PUFAs are also confirmed in vivo that both DHA and EPA trigger a reduction of VCAM-1 and ICAM-1 to inhibit PMN transepithelial migration in TNBS mice [44]. Notably, the excessive production of activated neutrophils not only eradicate invading pathogens but also cause extravascular tissue damage. The detrimental effect has been associated with an increased production of cytotoxic reactive oxygen and nitrogen species and lytic enzymes. This collateral damage can be avoided by treatment with n-3 PUFAs, which decreases the level of serum LTB4 released from neutrophils in UC patients [45]. In conclusion, both n-3 PUFAs and n-6 PUFAs could participate in the regulation of neutrophil infiltration, and n-3 PUFAs could avert the concomitant hurt caused by neutrophil production in IBD.

Dendritic cells (DCs) are an intermediate linker between the identified exogenous information and T lymphocytes, which is required for the attachment of ICAM-1 on DCs to lymphocyte function-associated antigen-1 (LFA-1) on T cells. Once major histocompatibility complex class-II (MHC-II) molecules on DCs bind to antigen receptors (TCRs) on T cells, accompanied by the combination of cofactors, such as CD80 and CD86 on DCs and CD28 and CTLA-4 on T cells, the antigen-related information will be conveyed. Many studies suggest that the functions of DCs can be modulated by LCFAs via adjusting these cell surface molecules. On the one hand, n-3 PUFAs could suppress the expression of CD69 and CTLA-4 on T lymphocytes that reduces DC immunity response [46, 47]. On the other hand, LCFAs were found to downregulate the MHC-II expression on intestinal DCs, thereby reducing the antigen-presenting ability of DCs [48]. In contrast, n-6 PUFA-derived PGE2 extends the level of costimulatory molecules both on DCs and T cell, including OX40 and CD70, and induces T cell proliferation [49, 50]. Therefore, it is easy to infer that the antigen presentation of DCs is protected by n-6 PUFAs series rather than n-3 PUFAs series. Kanai and Watanabe [51] posited that these findings shed light on the development of an implacable strategy to treat IBD. However, with different affinities to costimulatory factors, various LCFAs have different degrees of influence on DC function. Moreover, the effect of LCFAs on DC function varies from intestinal inflammatory conditions. Therefore, for LCFAs targeting DCs in IBD, these aspects need to be carefully explored in future trials.

B cells are another antigen-presenting cell type with unique secretory function. The key aspects of B cell function have been reported to be regulated by LCFAs in a steady accumulation of data. In terms of B cell activation, using palmitic acid, oleic acid, and n-3 PUFAs to deal with B cells for 48 hours, the CD69 expression of the activation marker of B cells is lowered more than 40% by palmitic acid and oleic acid, while it is not influenced by n-3 PUFAs [52]. However, n-3 PUFAs are demonstrated to influence the B cell lipid raft microdomain clustering to alter B cell function. Such altered organization of the lipid membrane keeps the line with B cell function by changing transmembrane signaling [53]. Furthermore, Gurzell et al. [54] identified this mechanism of n-3 PUFAs in a colitis-prone mouse model. After feeding mice with a diet rich in n-3 PUFAs for five weeks, they examined B cells extracted from the spleen and discovered modifying lipid composition on the B cell membrane, upregulating the activation marker of B cells, as well as arising fecal sIgA. The same function on intestinal sIgA is replicated in a palmitic acid diet [55]. As we mentioned above, sIgA is essential for the intestinal mucus barrier, whose hypersecretion contributes to pathogen defense in IBD. However, analysis from IBD patients demonstrates that intestinal B cells are likely to dominate pathogenic influence on intestinal immunity. Rectal mucosa of UC patients has shown increased B cell activation versus the healthy control [56]. Eosinophilic recruitment in IBD is also reported to take place owing to the accumulating chemokines brought by B cell activation [57]. Given the current gap in the reports about the functions of dietary n-3 PUFAs on B cells among IBD patients, the related studies should apply more focus on the bidirectional regulation of B cells on intestinal immunity in vivo.

T cells and their productions are documented in the pathogenesis of IBD. One type of T cell is CD4+ T cell, including T helper (Th) 1, Th2, Th17, and regulatory T cells (Tregs), collectively belonging to antigen-presenting cells. Another type of T cell is the terminal effector of antigen-presenting cells, CD8+ T cell [58]. LCFAs may involve in IBD pathogenesis via influencing these cell subsets. When using Helicobacter hepaticus to infect SMAD3−/− mice, an inflammatory colitis model, and then feeding these mice with dietary fish oil (DFO) for eight weeks, Woodworth et al. [59] detected a higher infiltration of the inflammatory cell on cecum and colon tissues than those just infected with H. hepaticus. Moreover, these mice even appear to display emerging dysplastic crypts and mitotic figures on colon and cecum tissues. Meanwhile, the consumption of DFO reduces the percentage of CD8+ cell, diminishing the expression of CD69 on CD4+ T cell and increasing the count of FoxP3+ CD25+ T cells. However, the results from other teams suggest that the role of n-3 PUFAs on colitis depends on inflammatory types and sites. These groups used n-3 PUFAs to deal with acute or chronic animal models of intestinal inflammation and found that there is no difference concerning the proportion of Th17 cells between colonic lamina propria and spleen in the acute model of intestinal inflammation. However, Th17 cells located in colonic mucosa express lower activated cytokines and higher suppressive cytokine in the chronic model of intestinal inflammation [60, 61]. Therefore, the series of n-3 PUFAs would be beneficial in chronic intestinal inflammation but could be harmful in acute intestinal inflammation. Regarding n-6 PUFAs, such dependent role was also implicated in the animal IBD model [62]. Moreover, reducing n-6 PUFA-derived eicosanoids decreases the percentage of Th17 cells in TNBS-induced colitis [63]. However, various ratios of LCFAs have different effects on Th/Treg balance in the IBD model [64]. Future studies should establish an optimum proportion of LCFA consumption for skewing T cell differentiation towards the production of the anti-inflammatory Treg cell subset, particularly in IBD patients.

### 3.3. Effects of LCFAs in the Inflammatory Resolving Phase

The inflammatory resolving phase is a transitional process from the inflammatory response stage to the inflammatory self-limiting stage. Once the transition fails to conduct, the inflammatory homeostasis will be disrupted, and negative physiologic sequelae will occur. Such is the case in IBD, a disease mediated by chronic intestinal inflammation leading to intestinal stenosis. A plethora of recent studies have shown that specialized proresolving lipid mediators (SPM) have pleiotropic actions in response to prevent excessive inflammatory events and promote recurrent tissue homeostasis in IBD (Table 3). n-3 PUFA-derived metabolites are precursors of most SPM, including resolvins (Rvs), protectins, and maresins (MaR), while lipoxin is derived from AA. Among them, Rvs are nominated as RvE and RvD, respectively, from EPA and DHA. These chemical mediators are served as the components of mediating resolution and are coupled with multiple capacities that block neutrophil trafficking, induce phagocytosis, and clear apoptotic cells [65]. Thus, the implications of PUFA-derived SPM could promote inflammatory resolving.

Preventing the entrance of PMN cells into inflammatory sites is one of the proresolving properties of SMP. RvE1 is described as a prohibitor to transendothelial migration of PMN as well as a promoter to IL-12 [66]. With a higher concentration than RvE1 in the human body, RvD2 is equivalent to inhabit PMN infiltration, which relies on the elevated expression of G protein-coupled receptor 18 (GPR18) on PMN [67]. This blocking role also occurs for lipoxin A4 (LXA4) by stopping transendothelial migration of PMN across the blood vessel endothelium along with promoting their clearance from inflammatory sites, which are facilitated by activating human LXA4 receptor (ALXR) to govern gene expression [68]. Additionally, MaR1 significantly reduces the PMN in inflammatory organs without altering PMN in peripheral blood, suggesting a crucial regulating role of MaR1 on PMN entry into inflammatory sites [69]. Taken together, SMP triggers the resolution program to combat the spread of inflammation by timely inhibiting PMN entrance. However, since SMP has protective properties for inflammation through binding to its receptors on PMN, further investigations are warranted to identify the exact receptors on PMN, and other novel receptors have yet to be explored.

Macrophage phagocytosis refers key components to clear apoptotic immune cells from the inflammatory region and to bring the inflamed intestine to tissue repairment and regeneration [70]. Resolvins from n-6 PUFAs as well as n-3 PUFAs not only support the phagocytosis of macrophages but also polarize macrophages towards M2 phenotype, a type of proresolution macrophages. Both resolvins intraperitoneally injected significantly ameliorate body weight loss, colon epithelial damage, and macrophage infiltration of the DSS colitis model [71]. The latest research delineates the SMP role on macrophage autophagy by treatment of murine and human macrophages with LXA4 and resolvin D1 (RvD1), which induces an obvious formation of autophagosomes and favors the fusion of the autophagosomes with lysosomes, thus attributing to phagocytosis of apoptotic macrophages [72]. Additionally, LXA4 and its analogs can clear the excessive infiltration of neutrophils via enhancing the monocyte-derived macrophage phagocytosis role on apoptotic neutrophils [73]. RvE1 strengthens macrophage efferocytosis of apoptotic PMN and additionally grants nonapoptotic PMN in lymph nodes and the spleen with phagocytosed zymosan [74]. Moreover, RvD1 is indicated to enhance macrophage efferocytosis by binding with either the lipoxin receptor or the orphan GPR32 on PMN [75]. LXA4, RvE1, and protectin D1 have collectively been verified to upregulate the expression of C–C chemokine receptor 5 on apoptotic neutrophils, which is related to blocking chemokine signaling [76]. To sum up, these results emphasize that LFAC derivatives may have therapeutic potential to orchestrate the elimination of sustained inflammation by stimulating the formation of autophagosomes and the phagocytosis of apoptotic PMNs. In consideration of the fact that autophagy is an important factor in the pathogenesis of IBD, LFAC derivatives aimed at autophagy represent alternative therapeutic approaches for this chronic disease.

Recently, the vast findings are evidenced to reveal that the potential mechanisms would be indispensable for SPM in animal models of IBD. In the progression of the disease with a mouse model of DSS-induced acute intestinal injury, the precursor of protectin D1 was presented with an increase over 3-fold in the recovery phase than its original level [77]. The DPA-derived protectin and resolvin were shown to dampen intestinal inflammation and leukocyte adhesion in the mouse model of colitis. The endothelial monolayer of the human intestine administered with DPA-derived D-series protectin and resolvin also had lower cell adhesion response to TNF-*α* challenge compared with controls [8]. A study administrated with aspirin-triggered RvD1 (AT-RvD1) and RvD2 reported a reduced generation of IL-1*β*, CXCL1, NF-*κ*B, VCAM-1, and ICAM-1 in DSS- and TNBS-induced colitis. Both AT-RvD1 and RvD2 exposure additionally decrease the disease activity index, improve intestinal pathological changes, and inhibit polymorphonuclear infiltration in both experimental colitis models [78]. Colitis models treated with DPA-derived maresin 1 by Marcon et al. [79] had also been demonstrated with significantly decreased levels of inflammatory cytokines, including IL-1*β*, IL-6, IFN-*γ*, and TNF-*α* in DSS-induced colitis protocol, as well as IL-1*β* and IL-6 in the TNBS-induced colitis protocol. Moreover, in LPS-stimulated bone marrow-derived macrophage, MaR1 provides significant protection against neutrophil migration and reactive oxygen species production by upregulating mannose receptor C, type 1 mRNA expression. For these reasons, the SPM from n-3 PUFAs and n-6 PUFAs promotes an inflammatory resolving milieu, which provides useful alternative therapeutic approaches to control chronic inflammation in IBD. In the subsequent studies, expanding our understanding of resolving molecules in IBD patients are warranted to be performed in the future.

## 4. Fatty Acid Receptors as Drug Targets for IBD

A steady accumulation of studies shows the strong relationship between fatty acids (FAs) and specific receptor proteins, G protein-coupled receptors (GPCRs). The FA-related GPCRs were previously named as GPR40, GPR43, GPR41, and GPR120, correspondingly renamed to Free Fatty Acid receptor (FFA) 1, FFA2, FFA3, and FFA4 [80]. FFA2 and FFA3 are recognized by and respond to short-chain fatty acids (SCFAs), whereas FFA1 and FFA4 are activated by LCFAs. Moreover, GPR84 has been reported as another receptor for MCFAs [81]. Although a number of FFA receptor antagonists or agonists have been identified to date, only both GPR84 and FFA2 receptor antagonists have been taken into the clinical practice in IBD. GLPG0974, as the first FFA2 antagonist to treat IBD patients, reduces the neutrophil activation and infiltration in the phase I clinical trial [82] but shows no differences in clinical responses, histopathology scoring, and Mayo score in mild to moderate UC patients [83]. The GPR84 antagonist, GLPG1205, is also terminated in the phase II trial due to the same reason [83]. Additionally, the selective FFA1 and FFA4 agonists as pharmacological tools have mainly been developed and applied in diabetes [84, 85]. However, although the FFA1 and FFA4 are abounding in the intestine, especially in the colon [86], both agonists constituting unique and novel treatments for IBD have not been investigated. Furthermore, the modest ligand affinity of fatty acid agonists on its corresponding receptors obstructs their clinical application. Developing selective synthetic ligands to improve affinity and translating therapeutic targeting to yield real benefits for patients are imperative.

## 5. Conclusion

LCFAs have dual actions on intestinal inflammation in IBD by influencing the phage of pathogenic recognition and the infiltration and function of immune cells, along with the phage of inflammatory resolving. The mechanisms comprise the fact that LCFAs protect or dampen intestinal barriers, promote or inhibit TLR/NOD signaling pathways, and influence the balance between proinflammatory transcription factor NF-*κ*B and anti-inflammatory transcription factor PPAR-*γ*. Cumulative studies are utilizing LCFAs to access the remission of intestinal inflammation in IBD, both in IBD patient studies and animal experiments. Although the underlying signaling pathways have yet to be fully explored, the advantages of LCFA administration to facilitate the limitation of intestinal inflammation have been reflected by these studies. The aptitude of mucosal healing in IBD calls for development of new drugs. Administration of LCFAs should indeed be served as a useful therapeutic approach to treat IBD patients for its availability and effectivity.

## Figures and Tables

**Table 1:** Effects of LCFAs in the pathogen recognition phase in IBD.

Pathogen recognition phase	The type of LCFAs	Role
Intestinal barrier	DHA and EPA	Protect the tight junctions while reducing MUC2 secretion
	Palmitic acid and palm oil	Promote MUC2 secretion
	n-6 PUFAs	Reduce MUC2 secretion
TLR/NOD pathway	n-3 PUFAs	Upregulate TLR-2 gene
	n-9 PUFAs	Upregulate TLR-4 gene
	ALA	Inhibit the mRNA expression of TLR4, downregulate proinflammatory cytokines, upregulate anti-inflammatory cytokines, and decrease the mRNA expression of NOD
NF-*κ*B pathway	Fish oil	Downregulate the NF-*κ*B pathway
	ALA, EPA, and DHA	Downregulate the NF-*κ*B pathway
	Oleic acid	Downregulate the NF-*κ*B pathway
PPAR-*γ* pathway	Conjugated linoleic acid	Upregulate the PPAR-*γ* pathway
	DHA and EPA	Upregulate the PPAR-*γ* pathway

**Table 2:** Effects of LCFAs on immunity cells in IBD.

Effects of LCFAs on immune cells	The type of LCFAs	Role
Neutrophils	n-6 PUFAs	Inhibit neutrophil infiltration
	DHA and EPA	Inhibit neutrophil infiltration
	n-3 PUFAs	Avert the concomitant hurt caused by neutrophil production
Dendritic cells	n-3 PUFAs	Reduce the antigen-presenting ability of DCs
	PGE2	Promote the antigen-presenting ability of DCs
B cells	Palmitic acid and oleic acid	Reduce the immune response ability of B cells
	n-3 PUFAs	Reduce the immune response ability of B cells, promote intestinal sIgA secretion
T cells	DFO	Reduce the percentage of CD8+ cells, diminish the expression of CD69 on CD4+ T cells, and increase the count of FoxP3+ CD25+ T cells
	n-3 PUFAs	Reduce the activated cytokines of Th17 cells
	Eicosanoids	Decrease the percentage of Th17 cells

**Table 3:** Effects of LCFAs in the resolving phase in IBD.

Resolving phase	The type of LCFAs	Role
Neutrophil trafficking	RvE1, RvD2, LXA4, and MaR1	Inhabit PMN infiltration
Apoptosis and phagocytosis	Resolvins from n-6 and n-3 PUFAs	Promote phagocytosis of macrophages, polarize macrophages towards M2 phenotype
	RvD1	Promote phagocytosis of apoptotic macrophages
	LXA4	Promote phagocytosis of apoptotic macrophages and neutrophils
	RvE1	Promote phagocytosis of apoptotic neutrophils
	Protectin D1	Promote phagocytosis of apoptotic neutrophils
